# The Use of the Surgical Guide for Placing Miniscrew in Treatment of Class II Subdivision: A Case Report With 2-Year Follow-Up

**DOI:** 10.1155/crid/3082753

**Published:** 2025-06-24

**Authors:** Wenyong Liang

**Affiliations:** Ontario Dental Association, Toronto, Canada

**Keywords:** CAD/CAM, case report, Class II subdivision, miniscrew

## Abstract

**Background:** Treatment of Class II subdivision can present a challenge for the clinician because of its asymmetry and possible midline deviation. This case report documents the use of a computer-aided design/computer-aided manufacturing (CAD/CAM) surgical guide for miniscrew placement in a Class II subdivision treatment.

**Case Presentation:** A 17-year and 1-month-old female presented with a skeletal Class I relationship, but mild mandibular skeletal and dental midline shift to the right relative to the facial midline. A full-step Class II molar relationship on the right side and slight Class III molar relationship on the left side and a 4.0 mm deficiency of space in the maxillary were noticed. Using CAD/CAM technology, a surgical guide was designed virtually and 3D printed for predrilling. With the surgical guide, one ø1.3 mm twist drill was chosen to prepare a 4–5 mm deep hole in the alveolar process distobuccal to the maxillary right second premolar. A *ø*1.4 *mm* × 8.0 *mm* miniscrew was inserted into the prepared hole. With this miniscrew, the unilateral Class II relationship was corrected successfully by distalization of the unilateral maxillary dentition on the Class II side after 13 months of treatment.

**Conclusion:** Application of CAD/CAM surgical guide is very helpful for placement of the miniscrew. Class II subdivision may be treated by distalizing unilateral maxillary dentition on the Class II side using the miniscrew.

## 1. Introduction

Treatment of Class II subdivision can present a challenge for the clinician because of its asymmetry and possible midline deviation. Application of asymmetric headgear is one option, but the treatment outcome much relies on the patient's compliance [1]. Conventional approaches are usually inefficient at distalizing maxillary dentition. Maxillary molar extrusion and maxillary anterior teeth protrusion are often seen with unilateral tip-back mechanics [2]. Mandibular anterior teeth flaring and slight overcompensation of the original Class I side to Class III molar relationship are frequently noticed with Herbst appliance [3]. The mandibular incisors can be tipped labially with the Forsus device [4].

Molar distalization has become an efficient approach since temporary anchorage devices (TADs) including miniscrew and miniplate were brought into orthodontics. Unilateral Class II malocclusion is corrected by distalization of the maxillary molars with a miniscrew inserted in the interradicular space [5]. However, there is always the possible risk of trauma to the adjacent dental root during the placement of the miniscrew. Class II subdivision is treated using the unilateral zygoma-gear appliance supported by a zygomatic miniplate inserted on the Class II malocclusion side [6]. The disadvantage of this method is that the patient has to endure an invasive surgery.

With the CAD/CAM surgical guide, a miniscrew was safely inserted in the buccal alveolar process of the maxilla on the Class II side in a Class II subdivision case. The Class II subdivision malocclusion was corrected successfully by distalizing the unilateral maxillary dentition with the miniscrew.

## 2. Patient Information

A 17-year and 1-month-old female presented with a chief complaint of crooked teeth. Her medical and dental histories were noncontributory.

## 3. Clinical Findings

**Figure 1:**
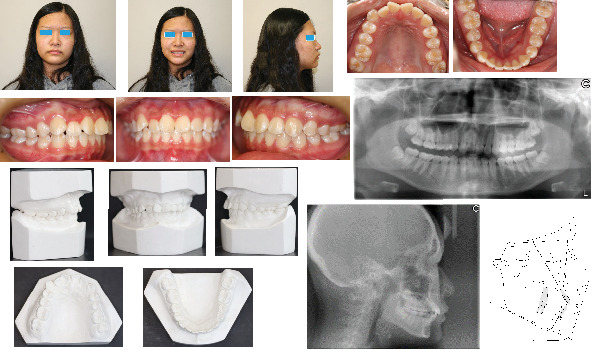
Pretreatment extra- and intraoral photographs, models, radiographs, and cephalometric tracing (black).

She had a slightly convex profile with mandibular skeletal and dental midline shift to the right by around 2.0 mm relative to the facial midline. The maxillary midline was coincident with the facial midline. The study model examination showed the maxillary and mandibular crowding 4.0 and 1.0 mm, respectively, a full-step Class II molar and canine relationship on the right side and a slight Class III molar but Class I canine relationship on the left side. The pretreatment cephalometric analysis revealed a skeletal Class I relationship but slight protrusion of bimaxillary incisors with U1/SN 116.0°, U1-NA 7.3 mm, and L1-NB 5.8 mm (Table 1). The pretreatment panoramic radiograph demonstrated the mild inclination of long axes of maxillary central incisors to the right and four wisdom teeth developing (Figure 1). No temporomandibular disorder (TMD) symptoms or signs and no centric occlusion and centric relation (CO/CR) shift were found clinically. The possible etiology for this patient's Class II subdivision is a combination of skeletal chin deviation toward the Class II side and mesial migration of the right maxillary dentition.

## 4. Diagnosis

The diagnosis was Class II subdivision malocclusion with mandibular skeletal and dental midline shift to the right.

## 5. Treatment

The treatment objectives were to correct the Class II and Class III molar relationships, solve bimaxillary crowding and mandibular bone and dental midline shifts, eliminate maxillary incisor protrusion, obtain normal overjet and overbite, and establish Class I molar and canine relationships.

The first treatment alternative was to extract the maxillary right second premolar for eliminating crowding, but the midline of the maxillary dentition would probably be deviated to the right. The second alternative was to distalize the unilateral maxillary dentition with one miniscrew to solve the unilateral Class II molar relationship. Interproximal trimming of the mandibular anterior teeth was a possible approach to the mandibular dental midline deviation. Orthognathic surgery may be necessary for the correction of the skeletal mandibular shift.

The patient and her parents chose the second alternative but refused to consider the tooth interproximal trimming and mandibular surgery.

**Figure 2:**
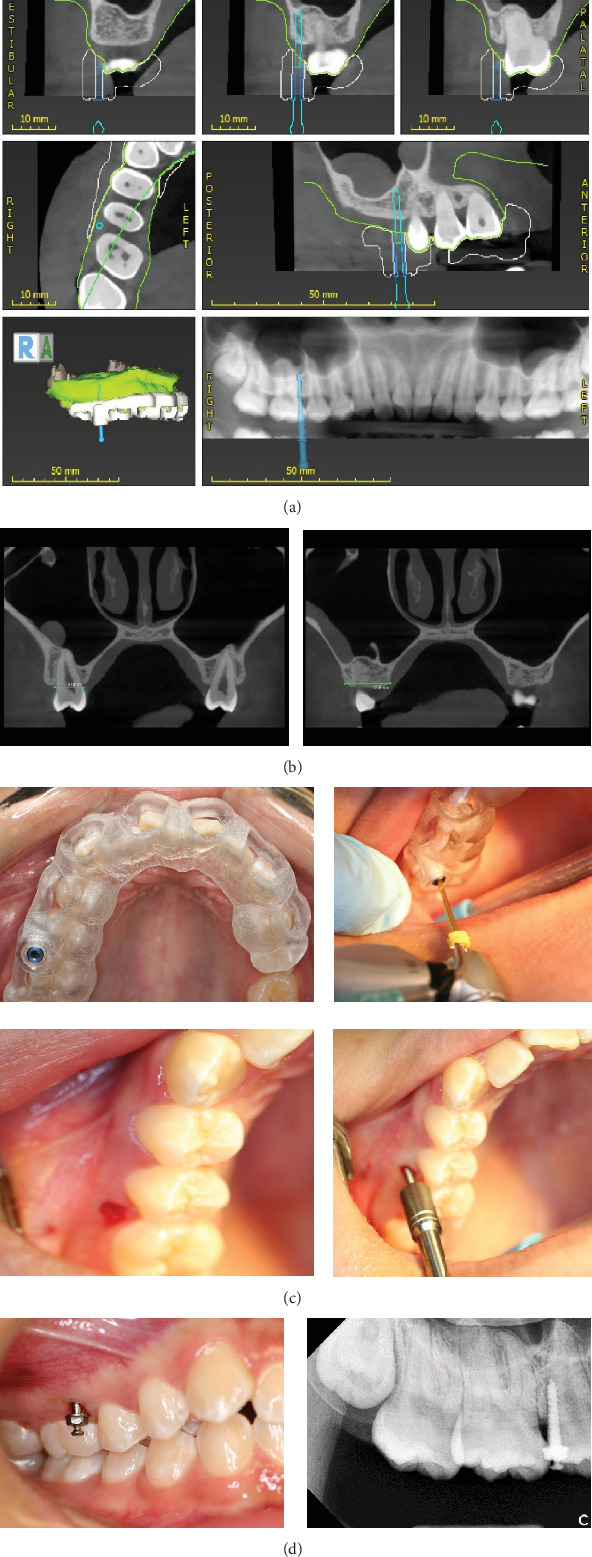
(a) Virtual planning with CAD/CAM technology, (b) widths of the maxillary second premolar and alveolar process, (c) predrilling with surgical guide and placement of miniscrew with a hand driver, and (d) the miniscrew parallel to the long axis of the maxillary second premolar.

As indicated in the previous study [7], a craniofacial cone-beam computed tomography (CBCT) (Kodak Dental Imaging Software 3D module V 2.4, protocol 80.25 × 80.25 × 50.1 *mm* at 120.0 kV, 8.0 mA, and 15.0 s) was scanned at the patient natural head position. The patient's stone model was scanned with a 3Shape scanner. The model scan file (STL) was merged with the CBCT file (DICOM file) using 3Shape Implant Studio software. The virtual planning was performed with a virtual pin of *ø*1.3 *mm* × 28.0 *mm*. The pin was placed close to the right maxillary first molar root but kept at least 1.0 mm distance in the buccal alveolar bone between the maxillary second premolar and first molar. It was kept almost parallel to the long axis of the maxillary right second premolar and 1.0 to 2.0 mm from the buccal aspect of the alveolar bone and at least 5.0 mm in depth in the alveolar bone without perforation of the maxillary sinus. The surgical template was 3D printed. Under local anesthesia, a Straumann ø1.3 mm twist drill was used to predrill a 4 to 5 mm deep hole in the alveolar process with the surgical guide. One *ø*1.4 *mm* × 8.0 *mm* miniscrew (made in Korea, Rocky Mountain Orthodontics, Denver, Colorado) was inserted into the prepared hole with a hand driver (Figure 2).

**Figure 3:**
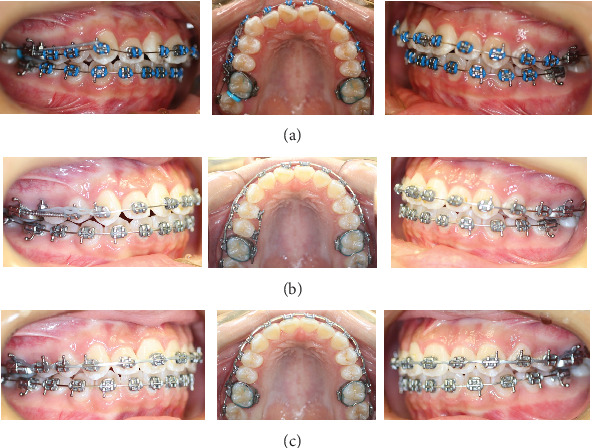
(a) Alignment and leveling commencing: (b) 8 months later and (c) 12 months later.

0.022″ slot brackets of the Roth prescription were chosen. The initial archwire was 0.014″ stainless steel then 0.016″, 0.018″, 0.016 × 0.022^″^, and 0.017 × 0.025 stainless steel archwires. A curved bend was made on the maxillary archwire to bypass the top of the miniscrew. One 0.010 × 0.030^″^ NITI open coil spring (150 to 200 g force) was placed on the maxillary archwire between the maxillary right second premolar and first molar. A power chain (200 g force) was placed from the miniscrew to the maxillary right canine and changed once a month (Figure 3).

**Figure 4:**
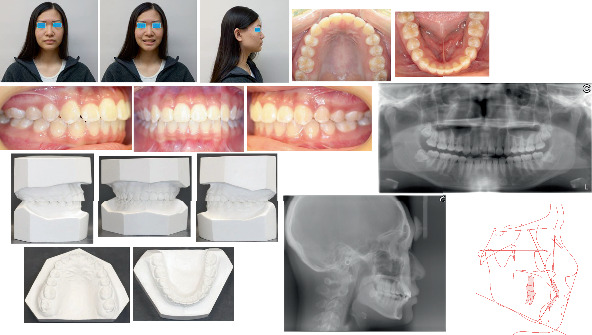
Posttreatment extra- and intraoral photographs, models, radiographs, and cephalometric tracing (red).

Three months later, the teeth leveling and aligning were improved. Class II traction on the right side with 5/16″ elastic was applied for 3 months in the hope of correcting the mandible and mandibular dental midline deviation. However, no improvement to the deviation appeared, so Class II traction was stopped. Eight months later, the bracket on the maxillary right second premolar touched the top of the miniscrew. Thus, the bracket was debonded, and two brackets were bonded onto the crown lingual surfaces of the maxillary right first and second premolars. A 0.017 × 0.025^″^ stainless steel sectional lingual archwire was placed. One longer 0.010 × 0.030^″^ NITI open coil spring was placed on the archwire between the maxillary right first premolar and first molar (Figure 3). Ten months later, Class I molar and canine relationships were established on the right side, so the miniscrew was removed, and the two lingual brackets and sectional wire were removed. One buccal bracket was rebonded on the right maxillary second premolar (Figure 3). One more month later, braces were removed, and retention was started with Essix retainers. The total duration of treatment was 13 months. The patient's profile was retained. The Class I molar relationship on the right side and Class I canine relationship on both sides were established. The right maxillary dentition was distalized by 3.5 mm measuring on the model. Mild Class III molar relationship was maintained on the left side. Normal overbite and overjet were formed, but the mandible and dental midline shift were not resolved. The posttreatment panoramic radiograph showed good axial inclinations of all teeth, but the two mandibular wisdom teeth mesial impact (Figure 4), so the patient was referred to see an oral surgeon for the four wisdom teeth extraction.

## 6. Follow-Up and Outcomes

**Figure 5:**
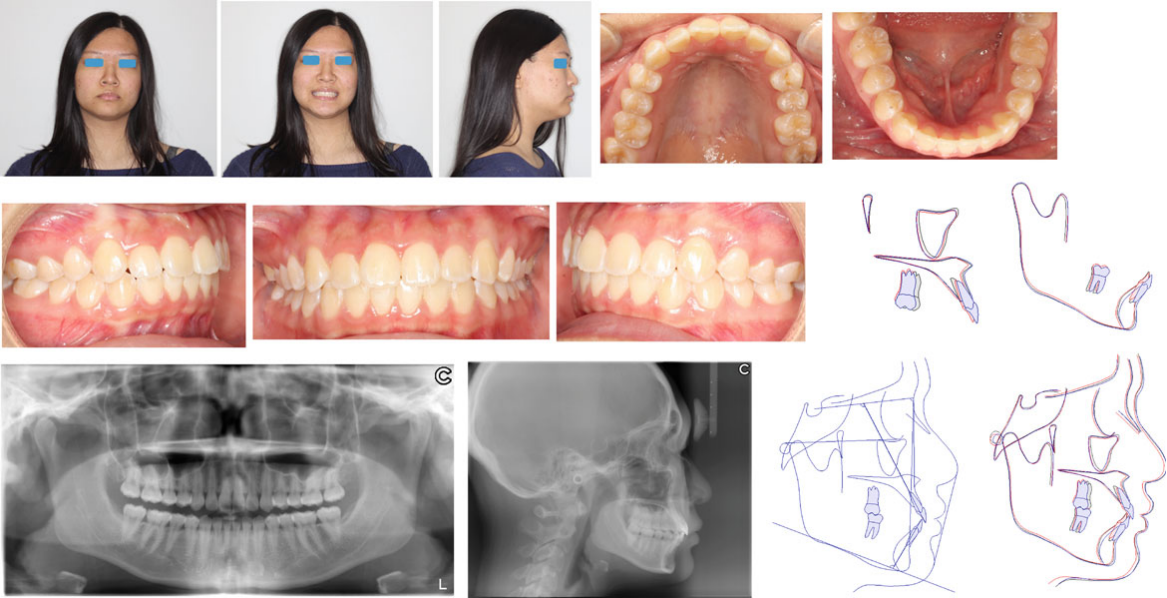
Two years posttreatment extra- and intraoral photographs, radiographs, cephalometric tracing (blue), and superimpositions of pre- (black), post- (red), and 2 years post-treatment (blue).

The treatment result was stable after 2 years post-treatment which was confirmed by extra- and intraoral examination, radiographs, and cephalometric analysis (Figure 5). The patient's four wisdom teeth have been extracted.

## 7. Discussion

Class II subdivision malocclusion is usually uneasy to deal with for the clinician. Unilateral maxillary premolar extraction treatment may result in a narrower and more posteriorly displaced arch form on the extraction side and the maxillary midline deviation to the extraction side [8]. Similarly, treatment with four or three premolar extractions (two maxillary premolars and one mandibular premolar on the Class I side) hardly avoids dental midline deviation [9]. About 50% of the Class II subdivision subjects have mandibular dental midline deviation from their facial midlines, and most of them exhibit some degree of mandibular skeletal asymmetry which makes the midline deviation correction very difficult [10]. In our case, the patient's mandible and dental midline shifted to the right, but the maxillary dental midline was coincident with the facial midline.

Class II subdivision is corrected by distalization of the maxillary dentition with a miniscrew inserted in the buccal interradicular space [5], but distalization is limited because the miniscrew is in the way of the distal movement of the maxillary second premolar. One solution is that first, the maxillary molars are distalized by placing a miniscrew in the interradicular space between the maxillary second premolar and first molar, then the anterior dental segment is retracted by replacing the miniscrew between the maxillary first and second molars [11], but the patient has to endure twice the insertion of miniscrews. The miniscrew placement may generate potential risks such as trauma to the dental root soft tissue injuries due to miniscrew slippage [12], and the proximity of a miniscrew to the root is a major risk factor for the failure of screw anchorage [13]. The maxillary teeth can be retracted by miniscrews placed in the infrazygomatic crest, but the placement of the miniscrew requires the clinician to possess much experience [14], and there is a significant difference among individuals in the depth and height of the infrazygomatic crest [15]. Class II subdivision is corrected by unilateral distalization of the maxillary dentition with a miniscrew-assisted palatal appliance [16], but it requires at least two miniscrews.

Class II subdivision was treated successfully by distalization of unilateral maxillary dentition with a *ø*1.4 *mm* × 8.0 *mm* miniscrew in our case. The surgical guide was designed and 3D printed with CAD/CAM technology so the miniscrew could be inserted accurately in the buccal alveolar process almost parallel to the long axis of the maxillary second premolar on the Class II side. A ø1.3 mm twist drill was used to prepare a 4 to 5 mm deep hole in the alveolar process for *ø*1.4 *mm* × 8.0 *mm* miniscrew. To obtain more primary stability, a ø1.5 mm or *ø*1.6 *mm* × 8.0 *mm* miniscrew can be chosen. The faciolingual cervix width of the maxillary second premolar and the buccal–lingual width of the alveolar crest at the site of the maxillary second premolar were around 8.0 and 12.8 mm, respectively (Figure 2). Thus, the miniscrew did not interfere with the distal movement of the right maxillary second premolar. However, the miniscrew did impede the buccal bracket on the maxillary second premolar when the tooth moved distally. This problem can be solved by tipping the miniscrew to the buccal by 15° to 30°.

## 8. Patient Perspective

The patient and her parents were very satisfied with the treatment results.

## 9. Informed Consent

The patient signed the consent for the publication of this case report and gave her informed assent.

## 10. Conclusions

Class II subdivision malocclusion may be treated by distalizing the unilateral maxillary dentition on the Class II side with the miniscrew. Using CAD/CAM technology, the surgical guide for predrilling can be designed virtually and 3D printed, so the miniscrew will be inserted into the prepared hole accurately and safely.

## Figures and Tables

**Table 1:** Cephalometric summary.

**Ceph name**	**Pretreatment**	**Posttreatment**	**2-Year posttreatment**	**Norm**	**Unit**
SNA	83.5	82.6	84.1	82 ± 3	°
SNB	81.8	81.1	82.0	80 ± 3	°
ANB	1.7	1.4	2.1	1.6 ± 1.5	°
SN/MP	29.1	30.6	27.3	32.9 ± 5.2	°
FH/MP	24.0	25.2	22.8	25.1 ± 4.5	°
U1/L1	118.8	121.5	118.0	130 ± 10	°
U1/SN^a^	116.0	108.8	110.9	103.4 ± 5.5	°
L1/MP	96.1	99.2	99.3	95 ± 7	°
U1-NA^a^	7.3	7.0	6.8	4.3 ± 2.7	mm
L1-NB^a^	5.8	6.6	7.2	4 ± 1.8	mm
UL-EL	−1.7	−2.5	−1.9	−2 ± 2	mm
LL-EL	−1.3	−1.2	−1.4	−2.8 ± 2	mm

Abbreviations: L1, mandibular central incisor; U1, maxillary central incisor.

^a^Significant deviation from a norm.

## Data Availability

The author can make the data available upon request.
